# New Genetic Lineage of Tula Hantavirus in *Microtus arvalis obscurus* in Eastern Kazakhstan

**DOI:** 10.2174/1874357900802010032

**Published:** 2008-04-03

**Authors:** Angelina Plyusnina, Juha Laakkonen, Jukka Niemimaa, Heikki Henttonen, Alexander Plyusnin

**Affiliations:** 1 Department of Virology, Haartman Institute, University of Helsinki, Finland; 2 Finnish Forest Research Institute, Vantaa research Unit, Finland

## Abstract

Genomic sequences of Tula (TULV) hantavirus were recovered from tissue samples of European common voles *Microtus arvalis* (subspecies *obscurus)* captured in Kazakhstan, Central Asia. Phylogenetic analysis of the S genomic segment of Kazakh TULV strains showed that they form distinct, well supported genetic lineage and share a more ancient common ancestor with two Russian lineages of TULV. The deduced sequence of the nucleocapsid (N) protein of Kazakh TULV strains carried specific amino acid signature: T274Q276T281. The *Microtus arvalis* group includes several sibling species and/or subspecies in Eurasia, indicating recent and ongoing evolutionary radiation. Our data on TULV lineages in Central Asia, the region not studied for hantaviruses earlier, highlight the diversity of both *Microtus* host and the virus and also their co-evolution.

## INTRODUCTION

Hantaviruses (genus *Hantavirus,* family *Bunyaviridae* are negative-strand RNA viruses with a tripartite genome [1]. Currently the genus consists of at least 22 distinct hantavirus species, each thought to be carried by a specific or closely related rodent or insectivore host. Some hantaviruses, e. g. Hantaan and Sin Nombre viruses, are severe human pathogens, while others, e. g. Tula virus (TULV), are apathogenic [2]. TULV was discovered in early 1990s, first as a sequence recovered from a tissue sample of European common vole, *Microtus arvalis* [3]. Later it has been isolated in cell culture [4], characterized, both genetically and antigenically [5-7] and since then used as a safe model to study hantavirus molecular organization and replication [7-11], hantavirus-host interactions [7, 11-19] as well as hantavirus genetics and evolution [20-23].

After its initial discovery in Central Russia (Tula region, approxinmately 100 km south of Moskow [3]), TULV was reported from several European countries: Czech Republic [5, 20], Slovakia [22], Austria [24], Belgium [25], Serbia [26], Croatia [27]; Germany [28], Poland [29], France and Switzerland [23]. There are also unpublished data (four S segment sequences deposited to the GenBank) on TULV from Omsk (West Siberia); so far, this has been the only example of TULV outside Europe. Here we present our findings on TULV in Eastern Kazakhstan, Central Asia, the region not studied for hantaviruses earlier.

## MATERIALS AND METHODOLOGY

### Rodent Tissue Samples

The *Microtus arvalis* group is taxonomically a difficult one, and the names and species status of taxa have often changed. Here we follow Wilson and Reeder, 2005 [30]. When necessary, we also give the formerly used scientific names. Altogether, 168 *Microtus* voles were trapped in four main regions in Eastern Kazakhstan between April 15 and May 3, 2003. These included 128 *M. arvalis obscurus,* 38 *M. socialis,* and two *M. oeconomus. *The trapping was done in surroundings of the cities Taldykorgan and Bakanas, on foothills up to the altitude 1200 m of Dzungarian Mountain range east of Taldykorgan, and close surroundings of Karatal plague field station. The details of trappings, study sites, rodent species and numbers captured, etc, are given in Henttonen *et al.* (in preparation). Briefly, animals captured with snap traps overnight were placed in a cold box, transferred to the laboratory and kept cold until dissected in the same day. Tissue samples from lung, kidney and spleen were fixed in RNAlater reagent (Ambion, Applied Biosystems) During the trapping time nights were still cold, even frosty, and rodents were in good shape when dissected. The RNAlater-fixed lung tissue samples were screened by immunoblotting for the presence of hantaviral nucleocapsid (N) protein antigen (Ag) as described before [20]. Briefly, the lung tissue samples (approximately 100mg) were homogenized by sonication in 500 mkl of Laemmli sample buffer. Aliquots of 10 μl were separated by electrophoresis in 10% sodium dodecyl sulphate-polyacrylamide gel and then blotted with rabbit polyclonal antibody raised against recombinant Puumala hantavirus N protein. Swine anti-rabbit antibodies conjugated with the horse radish peroxidase (Dako, Glostrup, Denmark) were used as secondary antibodies.

### Reverse Transcription - Polymerase Chain Reaction (RTPCR) and Sequencing

RNA was extracted from N-Ag-positive lung tissue samples using the TriPure RNA isolation system (Behringer Maannheim) following the manufacturer's instructions. PCR-amplicon corresponding to complete TULV S segment sequence was prepared as described earlier [3]. This product was cloned using pGEM-Teasy cloning system (Promega, Madison, WI) and sequenced automatically using the ABI PRISM^TM^ Dye Terminator or M13F and M13R Dye Primer sequencing kits (Perkin Elmer/ABI, NJ). Partial TULV S segment sequences were obtained by RT-nested PCRs. PCR-amplicons were gel-purified using QIAquick Gel Extraction kit (QIAGEN) and sequenced automatically using ABI PRISM^TM^ Dye Terminator sequencing kit.

### Phylogenetic Analysis

Multiple nucleotide alignments were prepared manually using the SeqApp 1.9a169 sequence-editing program. Phylogenetic analysis was performed using the PHYLIP program package [31] and TreePUZZLE [32]. In PHYLIP, 500 bootstrap replicates (SEQBOOT program) were fed to the distance matrice algorithm (DNADIST, with the ML model for nucleotide substitutions), distance matrices were analyzed with the Neighbor-joining (NJ, NEIGHBOR) or Fitch-Margoliash (FM, FITCH) tree-fitting algorithm; the bootstrap support values were calculated with the CONSENSE program. In TreePUZZLE, the Hasegawa-Kishino-Yano-85 model was used with 10,000 puzzling steps; base frequencies were estimated from the datasets. Hantavirus sequences used for comparison were recovered from the GenBank.

## RESULTS

### Trapping of Rodents and Screening of Rodent Tissue Samples

In the connection of a joint European Union supported research project on plague dynamics [33], also the material on rodent-borne viruses was collected in eastern Kazakhstan in spring 2003. Details of trapping and general results of screening for several rodent-borne viruses will be published elsewhere (Henttonen *et al.*, in preparation). One hundred twenty eight *Microtus* *arvalis* voles were first screened by immunoblotting for the presence of hantaviral N-Ag and 20 were found positive. Of those, 15 were from the surroundings of Taldykorgan, 1 from foothills of Dzungarian Range, and 4 from Karatal. In addition we had 38 *Microtus socialis* voles, 2 of which were weakly N-Ag -positive. Two *M. oeconomus* were N-Ag-negative. Next, the N-Ag-posive samples were analyzed using RT-PCR and hantaviral genomic sequences (S segment) were recovered from four of them (all four were *M. arvalis obscurus*).

### Genetic Characterization of Hantaviral Sequences

Since the PCR-amplicon corresponding to complete viral S segment sequence (1830 nucleotides, nt) was successfully obtained from only one sample (#322 from Karatal), a variety of RT-nested PCRs was used to recover partial S segment sequences from other three samples (experimental details are available upon request). Lower-than-usual efficiency of RT-PCR in detecting TULV sequences among the N-Ag-positive samples could be due to suboptimal conditions of field samples transportation and storage. All recovered hantaviral sequences belonged to TULV genotype. Corresponding wild-type TULV strains were designated as following: TUL/Karatal/Ma322/2003, TUL/Karatal/Ma340/2003, TUL/ Taldykorgan/Ma343/2003 and TUL/Taldykorgan/Ma216/ 2003, or Karatal322, Karatal340, Taldykorgan343, and Taldykorgan216, for short. For strains Karatal340 and Taldykorgan343, nt 142 to 1296 or nt 142 to 1206 of the S segment sequence were recovered, respectively. The shortest sequence, nt 909 to 1206, was recovered for strain Taldykorgan216. The S segment sequences of four Kazakh wt-TULV strains showed diversity between 1.6% and 2.4%. Notably, all observed nt substitutions appeared to be silent thus the deduced amino acid (aa) sequences of the N protein were identical in all four TULV strains. This suggested a strong negative (stabilizing) selection operating at the N protein level.

Complete S segment sequence of Karatal322 strain was 1830 nt long (the first and the last 22 nucleotides of the amplicon originated from the PCR primer and thus were not determined directly). The sequence included: the 5'-noncoding region (NCR, nt 1 to 42), the open reading frame for the 430 aa-long N protein (nt 43 to 1335) and the 3'-NCR (nt 1336 to 1830). The sequence showed the highest level of identity (87%) to TULV strains from Russia (Tula) and East Slovakia (Kosice). Other TULV strains from Slovakia (Malacky) and Russia (Omsk) and also strains from Chech Republic, Germany, Serbia, Croatia and Poland were more distantly related: sequence identities 84-86%. Similarly, the deduced aa sequence of the N protein from Karatal322 strain was most closely related to the N-sequences of Tula and Kosice strains: sequence diversity was as low as 1.9-2.8%. The N protein sequences of other TULV strains showed higher diversities: 3.0-4.7%. Kazakh N protein sequence carried specific aa signature, T274Q276T281, usually an indicator of a distinct lineage.

**Fig. (1):**
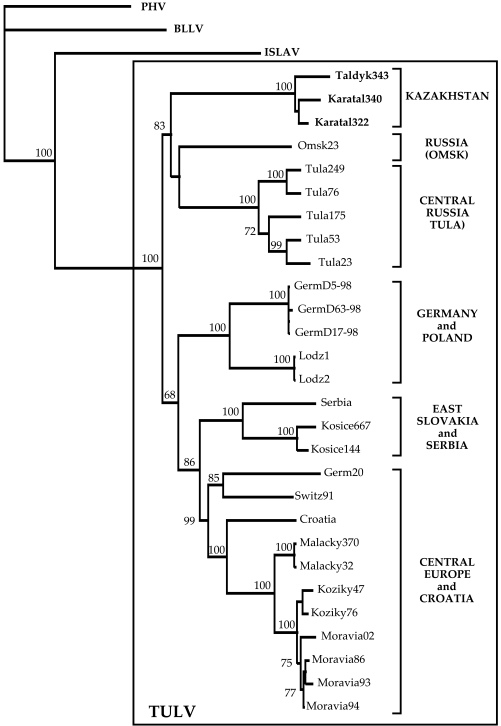
Phylogenetic tree (Neighbor-joining) of TULV based on the coding region of the S segment (nt 141-1206). Only bootstrap support values greater than 70% are shown. **PHV**, Prospect Hill virus, strain PH-1 (GenBank accession number Z49098); **BLLV**, Bloodland Lake virus, strain MO46 (U19303); **ISLAV**, Isla Vista virus, strain MC-SB-47 (U19302). Tula virus (**TULV**) strains: **Taldyk343**, Taldykorgan/Ma343/2003 (AM945879); **Karatal340**, Karatal/Ma340/2003 (AM945878); **Karatal322**, Karatal/Ma322/2003 (AM945877); **Omsk23**, MG23/Omsk (AF442621); **Tula 249**, Tula/249Mr/87 (Z30944); **Tula76**, Tula/76Ma/87 (Z30941); **Tula175**, Tula/175Ma/87 (Z30943); **Tula53**, Tula/53Ma/87 (Z30942); **Tula23**, Tula/23Ma/87 (Z30945); **GermD5-98**, Germany/D5-98 (AF289819); **GermD63-98**, Germany/D63-98 (AF289821); **GermD17-98**, Germany/D17-98 (AF289820); **Lodz1** (AF063892); **Lodz2** (AF063897); **Serbia** (AF017659); **Kosice667**, Kosice/667Ma/95 (Y13980); **Kosice144**, Kosice/144Ma/95 (Y13979); **Germ20**, Germany/g20-s (AF164093); **Switz91**, Switzerland/91Ma; **Croatia**, Croatia/c109-s (AF164094); **Malacky370**, Malacky/370Ma/94 (U31534); **Malacky32**, Malacky/32Ma/94 (Z48234); **Koziky47**, Koziky/5247Ma/94 (AJ223600); **Koziky76**, Koziky/5276Ma/94 (AJ223601); **Moravia02**, Moravia/5302Ma/94 (Z49915); **Moravia86**, Moravia/5286Ma/94 (Z48573); **Moravia93**, Moravia/5293Ma/94 (Z48574); **Moravia94**, Moravia/5294Ma/94 (Z48741).

On phylogenetic tree calculated for the S segment sequences, TULV strains from Kazakhstan formed distinct, well-supported genetic lineage indeed (Fig. (**1**); FM- and PUZZLE- trees revealed same branching pattern, not shown). Within the lineage, TULV strains show geographic clustering: two strains from Karatal were located close to each other. The monophily of these two strains was apparent on the NJ- and FM-trees and received reasonably high bootstrap support: 65% and 63%, respectively. Five other lineages of TULV were seen on the phylogenetic tree (Fig. **1**). Two Russian lineages included strains from Tula (Central Russia) and Omsk (West Siberia), respectively. The fourth lineage consisted of strains from Germany and Poland, the fifth lineage of strains from East Slovakia and Serbia, and the sixth - from Croatia and Central Europe (Germany, Switzerland, West Slovakia, and Czech Republic). Interestingly, the Kazakh lineage shared a more ancient common ancestor with two Russian lineages. Similarly, the fifth and the sixth lineages shared a common ancestor and, most likely, another, even more ancient common ancestor with the fourth lineage. The bootstrap support for the monophyly of these three lineages was 68%, for the NJ ree (Fig. **1**), 63%, for the FM-tree (not shown), and 60%, for the PUZZLE-tree (not shown).

With three exceptions, namely: (1) strain Tula249 from *M. levis (*former* rossiaemeridionalis *or* epiroticus),* a sibling species to *M. arvalis,* (2) strain Serbia from *M. subterraneus (*former *Pitymus* *subterraneus),* and (3) strain Omsk23 from *M. gregalis*, TULV strains presented on Fig. (**1**) originate from *M. arvalis.* The first strain belongs to Central Russian lineage and shows minimal genetic diversity from *M. arvalis-* carried strains that circulated within the same locality [3]. The second strain [26] is closely related to Kosice TULV strains from East Slovakia [22], with which it forms the fifth genetic lineage. Omsk23 strain presents a distinct lineage, but certainly belongs to TULV genotype.

## DISCUSSION

Data presented in this paper describe the first TULV strains from Central Asia. Kazakh strains from two localities, Taldykorgan and Karatal, constitute novel, distinct genetic lineage of TULV. This lineage is well-supported on phylogenetic trees and possesses a unique aa signature: T274Q276T281. Kazakh TULV lineage appears to be most closely related to lineages from Central Russia and Siberia. These three lineages share a common ancestor (bootstrap support value 83%, on the NJ-tree) and also several signature aa residues. Kazakh and Central Russian lineage share three signature aa residues: N42, E60, and I269, while Kazakh and Siberian lineage share one signature aa residue D291. These observations suggest common evolution history for Kazakh, Central Russian and Siberian lineages of TULV, which is somewhat different from the history of other three lineages that include strains from Central Europe (Germany, Switzerland, Slovakia, and Czech Republic), Poland and the Balkans and share another common ancestor. It should be noted that two groups of TULV lineages were seen in the recently published phylogeny based on partial S segment sequences [23] (except that Kazakh lineage was not present). The current phylogeny, based on longer sequences (almost complete coding region of the S segment), is more robust: most of the bootstrap support values are above the widely accepted confidential limit of 70% [34] - and hence more convincing.

Grouping of TULV lineages might had been rooted to glacial distribution patterns of *Microtus* voles across Europe and Central Asia. The ice age evolution of European small rodent species has been characterized by isolated refugia, not only in southern peninsulas, but also in more northern regions [35-37]. This is due to the fragmenting/isolating impact of several mountain ranges. In contrast, the last ice age and subsequent formation of vast steppe areas from Eastern Europe to Central Asia have probably supported more continuous *Microtus* populations.

As mentioned above, the vast majority of TULV genomic sequences available so far originate from *M. arvalis *that is considered the main natural host for the virus [1, 3]. There are also exceptions, like the three sequences listed in the Results and also, e.g., partial S-sequences recovered from *M. agrestis* (field vole) [27] and even from *Lagurus lagurus* (steppe lemming) (GenBank accession numbers AF442618-19). Geographical ranges of *M. levis, M. subterraneus* and *M. agrestis *overlap widely with the range of *M. arvalis* [38, 39] and therefore the virus spillover from *M. arvalis* to these species can not be excluded. However, some observations, e.g. higher infection rate in *M. agrestis* than in *M. arvalis *in Croatia, suggested, that *M. agrestis* in some areas can serve as another natural host for TULV [27]. As for *M. gregalis, *its range overlaps only with the easternmost parts of *M arvalis* range in western-central Siberia [39] and the spillover of TULV from *M arvalis* to *M. gregalis* is less likely. It thus seems that Tula can infect a number of *Microtus* species, even those in different subgenera [for subgenera, see 35]: *M. arvalis* and sibling species in *Microtus* group, *M. agrestis* in *Agricola* group, *M. subterraneus* in *Terricola* group, and even* M. gregalis* in *Stenocrarius* group that phylogenetically is most distant from the *arvalis* group [35]. In connection to this, it might be of interest that the carrier of Kazakh TULV strains, *M. arvalis obscurus*, currently classified as one of the chromosome forms in *M. arvalis* [30], has sometimes been considered a parapatric species of its own [38]. But, even if TULV proven to have two or more rodent host species, this would not be an exception. For instance, Sin Nombre hantavirus in some areas is harboured by deer mouse *Peromyscus maniculatus* and in others by white-footed mouse *P. leucopus* [40].

*Microtus* radiation in many taxa is an evolutionarily recent process, more recent than e.g. in *Myodes* (former *Clethrionomys*), and is still ongoing [35]. It seems that in Eurasia TULV can infect several *Micotus* species, also not closely related ones in different subgenera. Most importantly, TULV strains harboured by different *Microtus* species from the same region resemble each other. It therefore safe to assume that geographic distance was so far the major factor for the diversification of TULV lineages. Our data showing that Kazakh TULV strains are most closely connected to strains from West Siberia and Central Russia, be their host in the same or different subgenus of *Microtus*, support this point of view. Unusually high host diversity of TULV could be seen as an indication of young stage of its rodent host radiations and the virus-host co-evolution (the radiation of *Microtus* began 2 million years ago, but many of the sibling species have developed during the last 100 000 - 200 000 years).

## CONCLUSIONS

Our results demonstrate the presence of TULV in common vole *M. arvalis* in Kazakhstan. Kazakh TULV strains form a distinct genetic lineage and share a more ancient common ancestor with TULV strains currently circulating in Central Russia (Tula region) and West Siberia (Omsk region). The *Microtus arvalis* group includes several sister species and/or subspecies in Eurasia, indicating recent and ongoing evolutionary radiation. Our data on TULV lineages in Central Asia, the region not studied for hantaviruses earlier, highlight the diversity of *Microtus* host and the virus and their co-evolution.
